# Crystal structure and functional characterization of an isoaspartyl dipeptidase (*Cps*IadA) from *Colwellia psychrerythraea* strain 34H

**DOI:** 10.1371/journal.pone.0181705

**Published:** 2017-07-19

**Authors:** Sun-Ha Park, Chang Woo Lee, Sung Gu Lee, Seung Chul Shin, Hak Jun Kim, Hyun Park, Jun Hyuck Lee

**Affiliations:** 1 Unit of Polar Genomics, Korea Polar Research Institute, Incheon, Republic of Korea; 2 Department of Polar Sciences, University of Science and Technology, Incheon, Republic of Korea; 3 Department of Chemistry, Pukyong National University, Busan, Republic of Korea; Russian Academy of Medical Sciences, RUSSIAN FEDERATION

## Abstract

Isoaspartyl dipeptidase (IadA) is an enzyme that catalyzes the hydrolysis of an isoaspartyl dipeptide-like moiety, which can be inappropriately formed in proteins, between the β-carboxyl group side chain of Asp and the amino group of the following amino acid. Here, we have determined the structures of an isoaspartyl dipeptidase (*Cps*IadA) from *Colwellia psychrerythraea*, both ligand-free and that complexed with β-isoaspartyl lysine, at 1.85-Å and 2.33-Å resolution, respectively. In both structures, *Cps*IadA formed an octamer with two Zn ions in the active site. A structural comparison with *Escherichia coli* isoaspartyl dipeptidase (*Eco*IadA) revealed a major difference in the structure of the active site. For metal ion coordination, *Cps*IadA has a Glu166 residue in the active site, whereas *Eco*IadA has a post-translationally carbamylated-lysine 162 residue. Site-directed mutagenesis studies confirmed that the Glu166 residue is critical for *Cps*IadA enzymatic activity. This residue substitution from lysine to glutamate induces the protrusion of the β12-α8 loop into the active site to compensate for the loss of length of the side chain. In addition, the α3-β9 loop of *Cps*IadA adopts a different conformation compared to *Eco*IadA, which induces a change in the structure of the substrate-binding pocket. Despite *Cps*IadA having a different active-site residue composition and substrate-binding pocket, there is only a slight difference in *Cps*IadA substrate specificity compared with *Eco*IadA. Comparative sequence analysis classified IadA-containing bacteria and archaea into two groups based on the active-site residue composition, with Type I IadAs having a glutamate residue and Type II IadAs having a carbamylated-lysine residue. *Cps*IadA has maximal activity at pH 8–8.5 and 45°C, and was completely inactivated at 60°C. Despite being isolated from a psychrophilic bacteria, *Cps*IadA is thermostable probably owing to its octameric structure. This is the first conclusive description of the structure and properties of a Type I IadA.

## Introduction

Abnormally modified proteins containing a β-linked Asp residue (isoaspartyl linkage) are not allowed to accumulate in cells because the formation of isoaspartyl residues may induce the loss of protein function. Isoaspartyl-modified polypeptides result from chemical reactions such as the deamidation of asparagine or the dehydration of aspartate [1–3]. Bacteria contain three different enzyme recovery mechanisms that can repair isoaspartyl modified proteins. The first enzyme is protein-L-isoaspartyl O-methyltransferase (PIMT) [4–6]. PIMT repairs the damaged intermediate by transferring a methyl group from S-adenosyl-L-methionine to the α-carboxylate side chain of the isoaspartyl residue. The second enzyme is isoaspartyl aminopeptidase (IaaA) [7–9]. IaaA can hydrolyze the iosaspartyl peptide and has L-asparaginase activity. The third enzyme is isoaspartyl dipeptidase (IadA) [10, 11]. IadA is a binuclear metalloenzyme and a member of the amidohydrolase superfamily. This enzyme catalyzes the hydrolytic cleavage of β-aspartyl dipeptides. Thus, only IaaA and IadA can degrade isoaspartyl modified proteins because most proteases and peptidases do not recognize β-linked Asp residues [12, 13]. Without a specific dipeptidase, isoaspartyl dipeptides might accumulate and could be toxic to the cell.

In previous studies, the crystal structures of IadA from *Escherichia coli* (*Eco*IadA) have been solved both in its ligand-free state and complexed with aspartate, as well as with a transition state analog [the phosphinic inhibitor, Asp-psi(PO2CH2)-Leu-OH] and β-Asp-His [14–16]. These structural studies have demonstrated that the active site of *Eco*IadA contains two Zn ions, which are involved in the catalytic reaction. Structural information studies have also revealed conserved key residues in the catalytic site that constitute the metal and substrate binding sites. It is interesting to note that *Eco*IadA has a carbamylated lysine residue (K162) in its active site and this residue is important for metal ion coordination. The carbamylation on lysine is a post-translational modification that changes the charge on the lysine side chain from a positive to a negative charge and also extends the residue length by about 2 Å to allow for metal coordination. Further biochemical studies have provided insight into the substrate specificity of *Eco*IadA [14]. The results have shown that *Eco*IadA has a strong preference for β-Asp-Leu and β-Asp-Phe.

In the present study, we determined the crystal structures of IadA from *Colwellia psychrerythraea* strain 34H (*Cps*IadA) in its ligand-free form and that in complex with β-isoaspartyl lysine, and subsequently characterized its enzymatic properties. *Colwellia psychrerythraea* 34H is a strictly psychrophilic bacteria found in Arctic marine sediment and its full genome sequence information has been reported [17]. Thus, several proteins from the bacteria have been studied to examine cold-adapted activity and cold-active structural properties compared with mesophilic bacterial proteins. The most notable feature of *Cps*IadA is the different residue configurations in the active site compared with *Eco*IadA. In *Cps*IadA, the Glu166 residue corresponds in position to the carbamylated lysine 162 residue in *Eco*IadA. A comparison of the structures, together with extensive sequence alignment, indicated that IadAs can be classified into two groups, depending on the configuration of active site residues, as represented by *Eco*IadA and *Cps*IadA. In addition, the β-isoaspartyl lysine*-*complexed *Cps*IadA structure provided details of the interaction between β-isoaspartyl lysine and *Cps*IadA, as well as information about the conformational changes induced by substrate binding. Furthermore, biochemical analysis, together with the structural information and site-directed mutagenesis, also provided useful insights in understanding the substrate specificity of *Cps*IadA and as well as identifying residues important for enzymatic catalysis.

## Material and methods

### Cloning and mutagenesis of *Cps*IadA

*Colwellia psychrerythraea* strain 34H genomic DNA was isolated using a genomic DNA extraction kit according to the manufacturer’s instructions (Qiagen, Hilden, Germany) and then used as a template DNA for PCR amplification. The *CpsIadA* gene was amplified by PCR using primers 5′-AAGAAGGAGATATACCATGG GAAACGATAGCCAAACGATG-3′ (forward primer) and 5′-TGGTGGTGGTGGTGCTCGAG TTCGAATGTACCTTTAATCA-3′ (reverse primer), cut with NcoI and XhoI, followed by cloning into pET22b+ linearized with *NcoI* and *XhoI*. The underlined sequences represent the restriction sites for *NcoI* and *XhoI*, respectively. The resulting DNA in the expression vector contained a 6× His-tag at the C-terminus. The recombinant plasmid was confirmed by DNA sequencing (Macrogen, Daejeon, Korea), and then transformed into *E*.*coli* BL21 (DE3) for expression. Mutagenesis experiments were performed using a standard site-directed mutagenesis method. The mutagenesis primers used were: E80Q-forward, 5′- CATTACCGGTGGCGGCGGACAGGCAGGTTTTGCGACGCAAG-3′; E80Q-reverse, 5′- CTTGCGTCGCAAAACCTGCCTGTCCGCCGCCACCGGTAATG-3′; Y140F-forward, 5′-GTTGGACTGGTGGCTTCCACTTTCCTCTAAC-3′; Y140F-reverse, 5′- GTTAGAGGAAAGTGGAAGCCACCAGTCCAAC-3′; E166A-forward, 5′- GTTATTGGTATCGGAGCGTTTGCCATTAGTGATC-3′; E166A-reverse, 5′- GATCACTAATGGCAAACGCTCCGATACCAATAAC-3′; E166K-forward, 5′- CCCGTTATTGGTATCGGAAAGTTTGCCATTAGTGATC-3′; E166K-reverse, 5′- GATCACTAATGGCAAACTTTCCGATACCAATAACGGG-3′. The mutant PCR products were cloned and sequenced as described above.

### Expression and purification of *Cps*IadA

For the expression of the recombinant *Cps*IadA wild-type and mutant enzymes, the transformed *E*.*coli* BL21 (DE3) cells were grown in 2 L of Luria Bertani (LB) medium with ampicillin (100 μg/mL) at 37°C on a rotary shaker at 150 rpm until the OD_600_ reached 0.6–0.8. Isopropyl-1-thio-β-D-galctopyranoside (IPTG) was added to the medium at a final concentration of 0.5 mM. After incubation at 25°C, 150 rpm overnight, the cells were harvested by centrifugation, resuspended in lysis buffer (50 mM sodium phosphate, 300 mM NaCl, 5 mM imidazole pH 8.0), and disrupted by sonication. The cell lysate was centrifuged at16,000 rpm for 50 min (Vision VS24-SMTi V508A rotor), and the supernatants containing *Cps*IadA were loaded onto a Ni-NTA (Qiagen, Hilden, Germany) column. Recombinant *Cps*IadA bound to the Ni-NTA resin was washed with wash buffer (50 mM sodium phosphate, 300 mM NaCl, 20 mM imidazole pH 8.0) and eluted with elution buffer (50 mM sodium phosphate, 300 mM NaCl, 300 mM imidazole pH 8.0). The eluted *Cps*IadA was concentrated using Amicon Ultra-15 Centrifugal Filters (Ultracel-10 K; Merck Millipore Ltd., Country Cork, Ireland). Protein fractions containing *Cps*IadA were purified by gel filtration on a Superdex 200 column (GE Healthcare, Piscataway, NJ, USA) pre-equilibrated with 20 mM Tris-HCl (pH 8.0), 150 mM NaCl.

### Crystallization and data collection

Purified *Cps*IadA and *Cps*IadA E80Q mutant proteins were concentrated to 35 mg/mL and 54 mg/mL respectively. For determination of the enzyme-substrate complex, β-aspartyl lysine (5 mM) was added to the *Cps*IadA E80Q mutant protein solution and incubated for two hours at room temperature before crystallization. Initial screening of crystallization conditions was carried using a crystallization Mosquito robot (TTP Labtech, Cambridge, MA, USA) with the hanging-drop vapor-diffusion method at 293 K in 96-well crystallization plates (Emerald Bio, Bainbridge Island, WA,USA). The drops contained 0.6 μL of protein solution and 0.6 μL of reservoir solution and were equilibrated against 300 μL of reservoir solution. The successful crystallization conditions for *Cps*IadA consisted of 0.1 M Bis-Tris propane pH 7.0, 1.4 M sodium malonate (SaltRx #C11). The best crystals for the β-aspartyl lysine complexed *C*psIadA E80Q mutant appeared in 0.1 M Bis-Tris propane pH 7.0, 1 M ammonium citrate tribasic pH 7.0, (MCSG3 #C7). The crystals obtained were protected from the liquid-nitrogen gas stream using Paratone-N oil (Hampton Research, Aliso Viejo, CA, USA).

X-ray diffraction data were collected using the BL-5C beam line of the Pohang Accelerator Laboratory (PAL; Pohang, Korea). At a resolution of 1.85 Å, the data set for ligand-free wild type *Cps*IadA contained 100 images with 1° oscillation and an exposure time of 1 s per image and at a resolution of 2.33 Å the data set for β-aspartyl lysine complexed *C*psIadA E80Q mutant contained 100 images with 1° oscillation and an exposure time of 1 s per image. The data sets were indexed, processed and scaled using *HKL*-2000 program [18]. The statistics for the X-ray diffraction data sets are shown in Table 1.

**Table 1:** Data collection and refinement statistics.

Data set	*Cps*IadA	*Cps*IadA E80Q mutant complexed with β-isoaspartyl lysine
X-ray source	PAL 5C beam line	PAL 5C beam line
Space group	*P*42_1_2	*P*42_1_2
Wavelength (Å)	0.9796	0.9796
Resolution (Å)	50.00–1.85 (1.88–1.85)	50.00–2.33 (2.37–2.33)
Total reflections	554004	239212
Unique reflections	79057	38898
Redundancy	7.0 (7.1)	6.2 (6.7)
Completeness (%)	99.1 (100.0)	97.3 (100.0)
*R*_merge_^a^	0.087 (0.710)	0.143 (0.609)
Average I/σ (I)	42.1 (5.6)	30.2 (5.7)
CC_1/2_^b^	0.989 (0.943)	0.962 (0.894)
Refinement		
Resolution range (Å)	50.01–1.85 (1.90–1.85)	50.01–2.33 (2.39–2.33)
No. of reflections of working set	73450 (5392)	36407 (2705)
No. of reflections of test set	3814 (265)	1910 (138)
No. of amino acid residues	748	762
No. of water molecules	762	334
*R*_cryst_^c^	0.163 (0.236)	0.189 (0.237)
*R*_free_^d^	0.201 (0.286)	0.246 (0.320)
R.m.s. bond length (Å)	0.0223	0.0165
R.m.s. bond length (°)	2.1948	1.8402
Average B value (Å^2^) (protein)	25.262	40.209
Average B value (Å^2^) (solvent)	36.682	41.559

^a^ *R*_merge_ = ∑|<I>—I|/∑<I>.

^b^ CC_1/2_ values are the correlation between intensities from random half-data sets

^c^ *R*_cryst_ = ∑||Fo|—|Fc||/∑|Fo|.

^d^ *R*_free_ calculated with 5% of all reflections excluded from refinement stages using high-resolution data.

Values in parentheses refer to the highest resolution shells.

### Structure determination and refinement

The crystal structure of the ligand-free *Cps*IadA was solved by molecular replacement, using the *MOLREP* program from the CCP4 suite, with the crystal structure of IadA from *E*. *coli* (PDB code 1ONW; sequence identity, 43%) as the search model [15, 19]. The Matthew coefficient of 2.67 Å^3^Da^−1^ suggests that two monomers are present in the asymmetric unit [20]. The model was rebuilt using *Coot* and was refined with *REFMAC* from the CCP4 suite and the *PHENIX* program [21–24]. The final model of ligand-free *Cps*IadA had an *R*_work_ and an *R*_free_ of 16.3% and 20.1%, respectively. Molecular replacement for the β-aspartyl lysine complexed *Cps*IadA E80Q mutant was conducted using the *MOLREP* program from the CCP4 suite with the final refined crystal structure of ligand-free *Cps*IadA as the search model. Successive rebuild and refinement were performed using the *REFMAC* program from the CCP4 suite and the *PHENIX* program. The chemical coordination file for β-aspartyl lysine was built using *Coot* and eLBOW [25]. The final model of the β-aspartyl lysine complexed *Cps*IadA E80Q mutant had an *R*_work_ and an *R*_free_ of 18.9% and 24.6%, respectively. The qualities of the final structural models were checked using *MolProbity* [26]. The detailed refinement statistics are listed in Table 1. The atomic coordinates and structure factors for ligand-free *Cps*IadA and the β-isoaspartyl lysine bound *Cps*IadA E80Q mutant have been deposited in the Protein Data Bank (http://www.rcsb.org/) under accession codes 5XGW and 5XGX, respectively.

### Analytical ultracentrifugation

Sedimentation velocity analysis of *Cps*IadA was performed at 20°C with a XL-A analytical ultracentrifuge (Beckman Coulter, Brea, CA, USA). The protein solution (0.5 mg/mL) was dissolved in a buffer of 25 mM Tris-HCl (pH 8.0), 200 mM NaCl, 5 mM MgCl_2_ and 2 mM dithiothreitol. The sample and reference sectors of the dual-sector epon centerpiece were filled with the *Cps*IadA protein solution and the buffer, respectively, and the cell was centrifuged at a rotor speed of 45,000 rpm. The sedimentation profile was monitored over time at 280 nm, and the experimental data were analyzed using the SEDFIT program [27, 28].

### Enzyme activity assay

The activity of *Cps*IadA was assayed using a continuous spectrophotometric coupled enzyme assay, as previously described [14]. Aspartyl dipeptides including β-Asp-Leu, β-Asp-Gly, β-Asp-Phe, β-Asp-Lys, and β-Asp-His, and α-Asp-Leu were tested as substrates. The standard reaction was carried out at 30°C and the reaction mixture contained 100 mM HEPES (pH 8.0), 100 mM KCl, 3.7 mM α-ketoglutarate, 0.4 mM NADH, 0.64 unit of malate dehydrogenase, 6 units of aspartate aminotransferase, and *Cps*IadA. The reaction was initiated by the addition of 10 mM substrate. The hydrolysis of aspartyl dipeptides was monitored by coupling the formation of aspartate to the oxidation of NADH. The change in the NADH concentration was measured at 340 nm (Multiskan GO, Thermo Scientific, Vantaa, Finland). The activity of *Cps*IadA toward β-Ala-Ala was assayed using alanine dehydrogenase [14]. The reaction mixture contained 100 mM HEPES (pH 8.0), 1.5 mM *p*-iodonitrotetrazolium violet (INT), 1.5 mM NAD^+^, 2.0 units of diaphorase, 7 units of L-alanine dehydrogenase, substrate, and *Cps*IadA. The conversion of INT from the oxidized to the reduced form at 30°C was monitored at 500 nm.

### Temperature and pH studies

To investigate the effect of reaction temperature on the activity of *Cps*IadA, reactions were performed at temperatures over a range of 5 to 60°C under the standard assay conditions, using β-Asp-Leu as a substrate. To evaluate the thermal stability of *Cps*IadA, the enzyme solution was incubated at 0, 20, 50, 60, and 70°C for 90 min, and aliquots were taken every 15 min. The residual activities were measured under the standard assay conditions. To determine pH dependence of *Cps*IadA, reactions were carried out at various pHs ranging from 5.0 to 10.0. The various buffers used were 100 mM sodium acetate (pH 5.0–7.0), 100 mM HEPES (pH 6.5–8.5), and 100 mM Tris HCl (pH 7.0–10.0).

### Kinetics

The kinetic parameters of the wild-type enzyme were determined in the same reaction mixtures as described above by varying the concentration of the dipeptide substrate. The data were fit to the Michaelis-Menten equation using a nonlinear regression (GraphPad Prism 5 Software, San Diego, CA, USA).

### Circular dichroism

Circular dichroism (CD) measurements were performed using a Chirascan Circular Dichroism Spectropolarimeter (Applied Photophysics, Surrey, UK) equipped with a temperature control system. The CD spectra of samples in a quartz cuvette with a 0.1 cm path length were recorded in the far-UV region (190 to 260 nm). Scans were collected at 0.1-nm intervals with a 1-nm bandwidth. Each spectrum was corrected in triplicate, followed by averaging, and baseline subtraction. The thermal denaturation curve was obtained by plotting changes in the CD values at 222 nm over a temperature range of 5 to 99°C at intervals of 2°C. The denaturation temperature (*T*_m_) was defined as the point at which 50% of the sample denatured.

### Phylogenetic tree

For phylogenetic analysis of *Cps*IadA, bacteria and archaea sequences homologous to *Cps*IadA with at least 40% of amino acid sequence identity were used. The analysis involved a total of thirty-nine amino acid sequences including *Cps*IadA. Multiple sequence alignment was carried out using Clustal Omega [29] with default parameters. Based on the alignment, a phylogenetic tree was drawn using the Neighbor Joining method in MEGA7 [30].

## Results and discussion

### Overall structures of ligand-free and β-isoaspartyl lysine-bound *Cps*IadA

**Fig 1:**
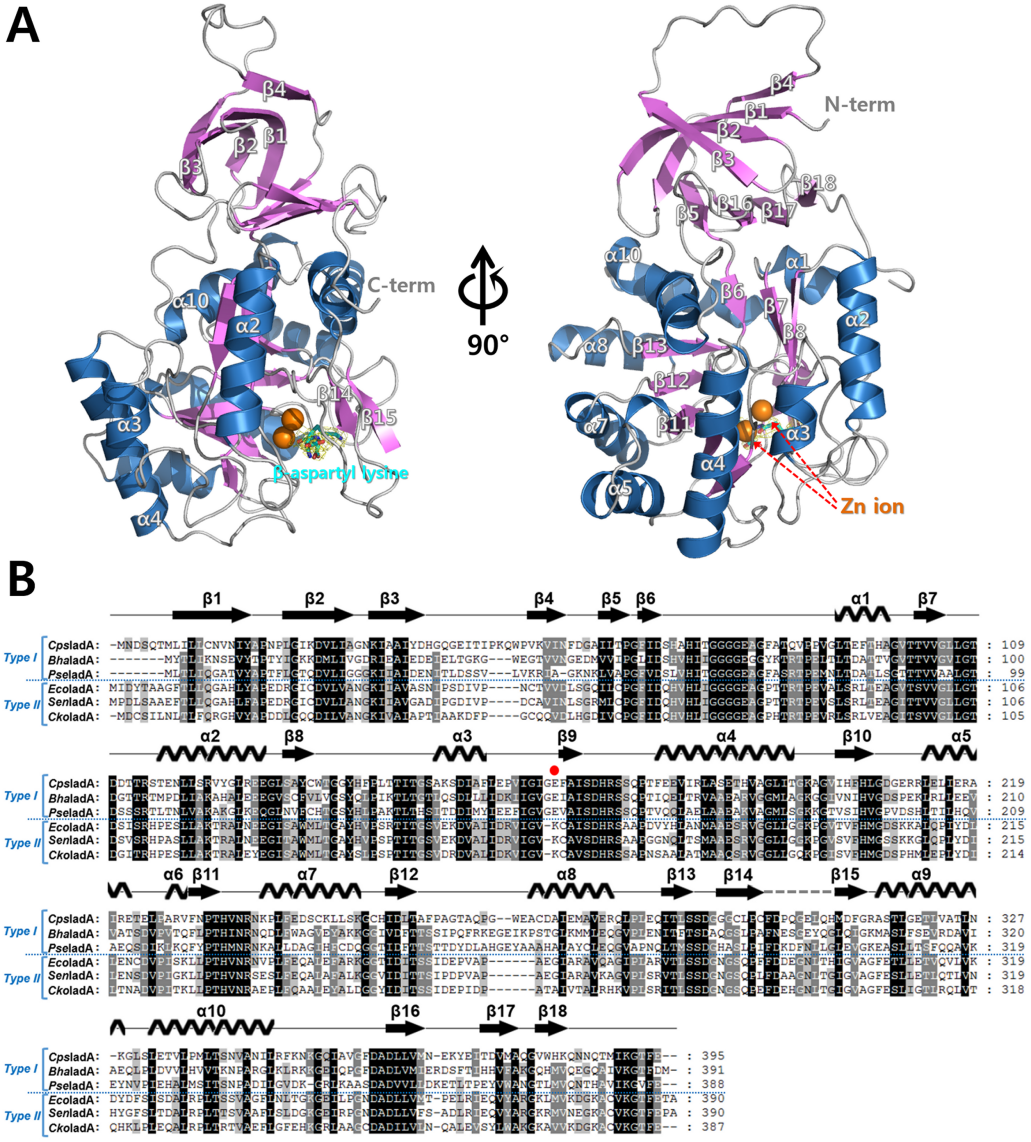
Crystal structure of *Cps*IadA and multiple sequence alignment with representative Type I IadAs and Type II IadAs. (A) Ribbon diagram showing the overall structure of the β-isoaspartyl lysine complexed *Cps*IadA. The monomer of *Cps*IadA is composed of two separate domains: the β-sandwich domain and the catalytic domain. The β-sandwich domain contains both the N- and C-termini and they are labeled as such. Two Zn ions are bound in the catalytic domain and these are indicated by the orange color. (B) Multiple sequence alignment of representative Type I IadAs and Type II IadAs. Type I IadAs have a glutamate residue for metal binding whereas Type II IadAs contain a carbamylated-lysine residue instead of a glutamate residue at the corresponding site. The Glu166 residue of *Cps*IadA is indicated with a red circle. The secondary structures obtained from the crystal structure of the β-isoaspartyl lysine complexed *Cps*IadA are shown above the aligned sequences. The disordered region is represented with a grey dashed line. The aligned sequences include *Cps*IadA (UniProtKB code Q484B6), *Bha*IadA (UniProtKB code Q9KDT2), *Pse*IadA (UniProtKB code E6RGG2), *Eco*IadA (UniProtKB code P39377), *Sen*IadA (UniProtKB code Q8Z0X6), and *Cko*IadA (UniProtKB code A8ADA6).

The ligand-free crystal structure of isoaspartyl dipeptidase from *Colwellia psychrerythraea* 34H (*Cps*IadA) and structure of the *Cps*IadA E80Q mutant complexed with β-isoaspartyl lysine were determined to resolutions of 1.85 Å and 2.33 Å, respectively (Fig 1 and Table 1). Both structures belong to the *P*42_1_2 space group and contain two monomers in the asymmetric unit. Those two monomers form a dimer, and four dimers from neighboring the asymmetric unit form a propeller shaped octameric structure. The *Cps*IadA monomer structure contains ten α-helices and eighteen β-strands, and the overall structure is divided into two distinct domains (a β-sandwich domain and a catalytic domain) (Fig 1A). The β-sandwich domain is composed of eight β-strands (β1, β2, β3, β4, β5, β16, β17, and β18) and this domain contains both the N-and C-termini. The catalytic domain forms an αβ-barrel structure with the eight central β-strands surrounded by the ten α-helices. Two Zn ions are located in the central cavity of the αβ-barrel structure.

Although no exogenous metal ions were added to the *Cps*IadA protein solution during purification or crystallization, two strong electron densities were identified in the Fo-Fc map. A careful examination of the interactions and comparison with previously determined *Eco*IadA structure (which contained two Zn ions at corresponding positions) indicated the potential for two divalent metal ions. We performed an X-ray fluorescence scan using the crystal to determine the identity of the metal. The result showed that a strong fluorescence signal was detected near the zinc K absorption edge and that the emission peaks from this scan were unambiguously characteristic for zinc. Therefore, we confirmed the presence of Zn ions. Zinc ions were, therefore, included in the model. After refinement, the B-factors of ZN1 and ZN2 on chain A were 31.90 Å^2^ and 24.13 Å^2^, respectively (the overall B-factor of protein residues is 25.3). Moreover, the occupancies of the two Zn ions have been estimated as 1.0 using the Phenix program [24], which further explains their incorporation (and retention) within the protein structure without the addition of exogenous Zn ions (S1 and S2 Figs).

Previous studies have shown that these metal ions are directly involved in the catalytic mechanism of IadA [14, 15]. The antiparallel β14 and β15 strands are located near the metal binding site and appear to form the gate for substrate entry or product release because of the relatively high B-factors observed in this region. In the crystal structure of the ligand-free *Cps*IadA, this part of the β14 and β15 region (residues 297–312) was disordered. In the case of the crystal structure of the *Cps*IadA E80Q mutant complexed with β-isoaspartyl lysine, this region (residues 301–309) was also partially disordered.

Previously, isoaspartyl dipeptidase from *Escherichia coli* (*Eco*IadA) has been shown to be an octamer (tetramer of dimers) [16]. Consistent with this observation, analytical size-exclusion chromatography (SEC) and analytical ultracentrifugation (AUC) of *Cps*IadA showed that *Cps*IadA also adopted an octameric state in solution. AUC experiments using 0.5 mg/mL *Cps*IadA (residues 1–395; calculated molecular weight of 42.7 kDa for the polypeptide chain) gives a mass of 314 kDa (sedimentation coefficient of 13.37 S and a frictional ratio of 1.245), indicating that *Cps*IadA is a stable octamer in solution.

**Fig 2:**
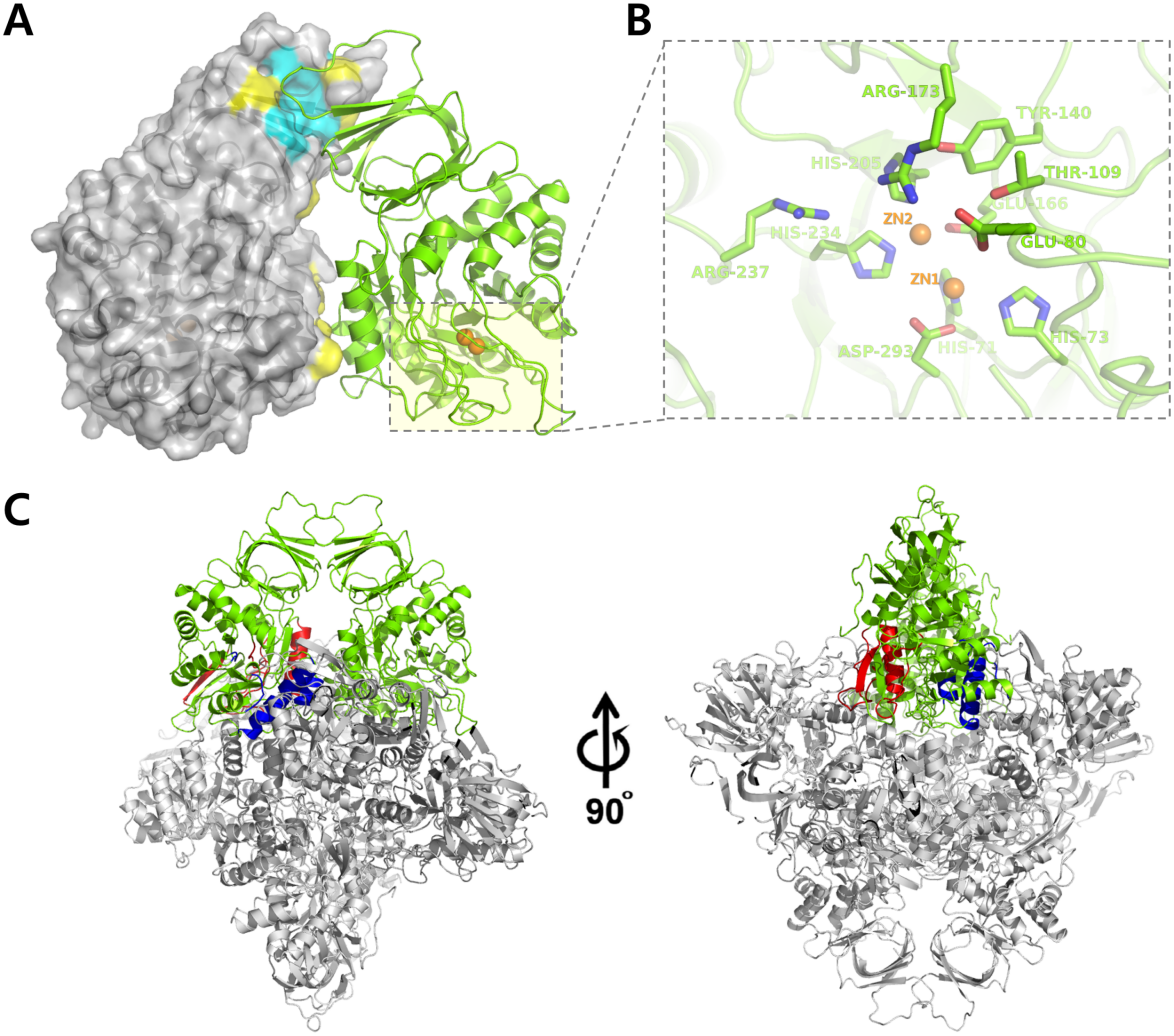
Octameric assembly of *Cps*IadA. (A) The asymmetric unit in the ligand-free *Cps*IadA crystal contains two *Cps*IadA molecules and this dimer is shown as two different colors (gray surface and green ribbon). The dimer interface is indicated by the yellow and green colors. (B) Close-up view of the active site of *Cps*IadA. Key active site residues and two Zn ions (ZN1 and ZN2; orange sphere) in the ligand-free *Cps*IadA structure are shown. (C) Ribbon representation of the octameric structure of ligand-free *Cps*IadA. The octameric structure of ligand-free *Cps*IadA is composed of a dimer of tetramers (one of the dimers is colored in green). The dimer-dimer interface is formed by two different patches colored in red and blue.

The *Cps*IadA monomer forms a dimer via strong hydrophobic interactions between each β-sandwich domain. The Trp51 residue located on the β3-β4 loop, protrudes toward the hydrophobic core (Leu8, Leu10, Leu28, Ala35, Ala36, Ile45, Ile47, and Val53; these residues are shown in cyan in Fig 2A of the other monomer. Several other hydrophobic interactions and hydrogen bonds also participate in dimerization (Asn32, Tyr38, Pro48, Pro52, Leu120, Ile147, Phe157, Leu158, and Tyr124; these residues are shown in yellow in Fig 2A. The dimer-dimer interface is formed by two different patches. In the first patch (shown in blue in Fig 2C), the α3 and α4 helices, the α5-α6 loop, and the α7-β12 loop regions interact with the α4 helix, the β6-α1 loop, the β7-α2 loop, the β8-α3 loop, the β10-α5 loop regions of the other dimer. The second interaction patch (shown in red in Fig 2C) is formed by the interactions between the β6-α1 loop, the β7-α2 loop, the α2 helix, the β15 strand and the C-terminal region of the neighboring dimer.

### *Cps*IadA active site

The *Cps*IadA active site contains two Zn ions (ZN1 and ZN2) with the ions being separated by 3.2 Å in distance. ZN1 interacts with the NE2 atom of His73, the OE2 atom of Glu166, and the OD1 atom of Asp293. ZN2 interacts with the OE1 atom of Glu166, the ND1 atom of His205, and the NE2 atom of His234 (Fig 2B). Notably, Glu166 directly interacts with the Zn ions in the *Cps*IadA structure. However, in the *Eco*IadA structure, carbamylated lysine 162 residue is located in this corresponding position and stabilizes the Zn ions. Further sequence alignment revealed that IadA containing bacteria and archaea can be classified into two groups (Type I IadAs having a glutamate and Type II IadAs having a carbamylated-lysine residue) based on the active site residue composition (Fig 1B).

**Fig 3:**
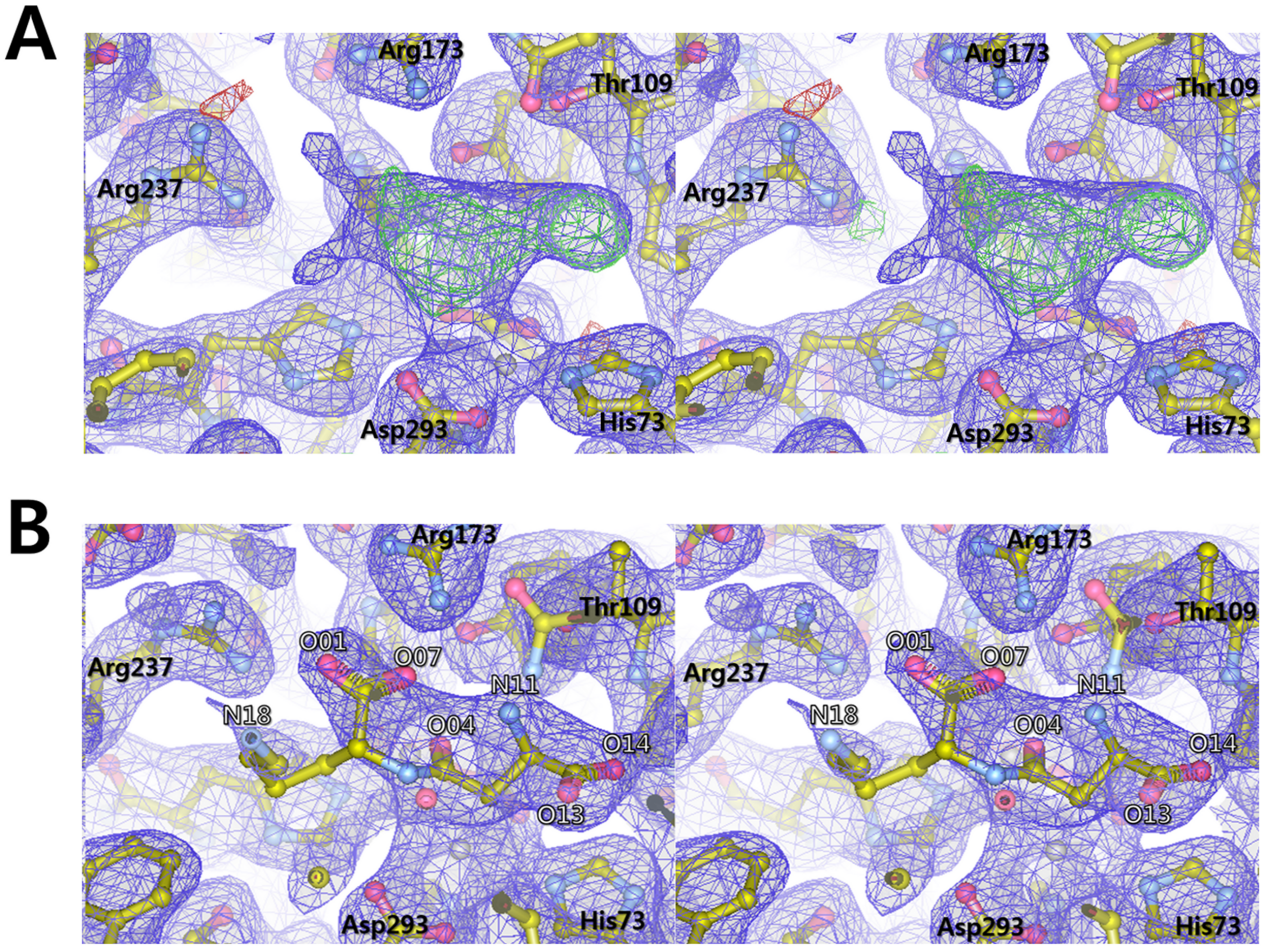
Stereo views of the β-isoaspartyl lysine binding site and binding mode in *Cps*IadA. (A) The Fo-Fc omit map (green, contoured at 3.0 σ) and 2Fo-Fc electron density maps (blue, contoured at 0.8σ) around the substrate binding site in *Cps*IadA are presented. (B) Refined 2Fo-Fc electron density maps (contoured at 0.8σ) with bound β-isoaspartyl lysine molecules are shown in the blue mesh. Carbon, oxygen, and nitrogen atoms are represented by sticks and colored yellow, red, and blue, respectively.

**Fig 4:**
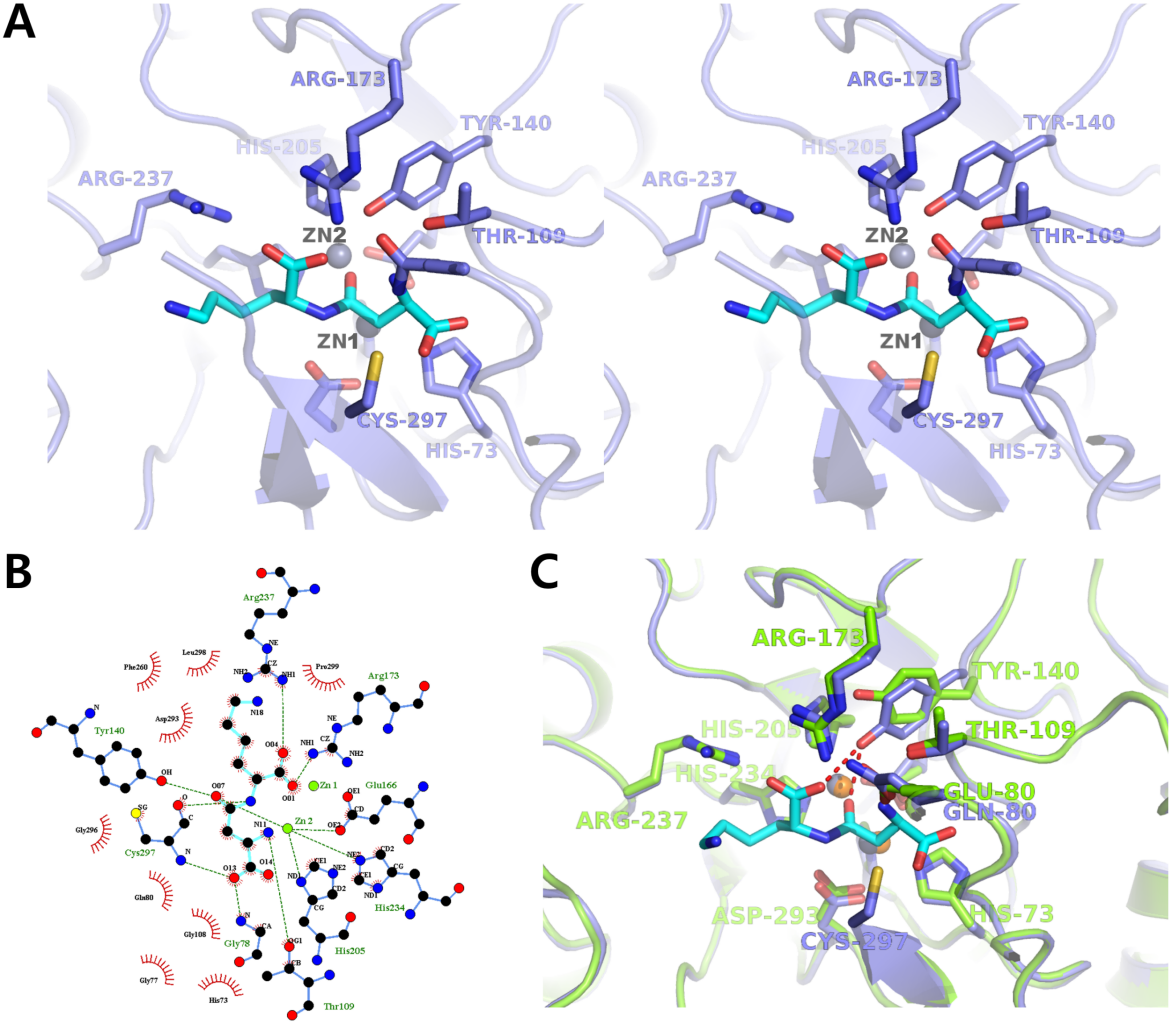
Active site of *Cps*IadA. (A) Stereo view of the active site structure of the *Cps*IadA E80Q mutant (slate blue) complexed with β-isoaspartyl lysine (cyan). (B) The bound β-isoaspartyl lysine molecule (cyan) and the binding interactions (hydrogen bonds are presented by green dashed lines and hydrophobic contacts are shown by red semi-circles) and are visualized using the Ligplot program. (C) Structural superposition and comparison of active sites between un-liganded *Cps*IadA (green) and the β-isoaspartyl lysine (cyan) bound *Cps*IadA (slate blue). Zn ions in *Cps*IadA are represented by orange spheres and the Zn ions in β-isoaspartyl lysine bound *Cps*IadA have been colored in gray.

**Fig 5:**
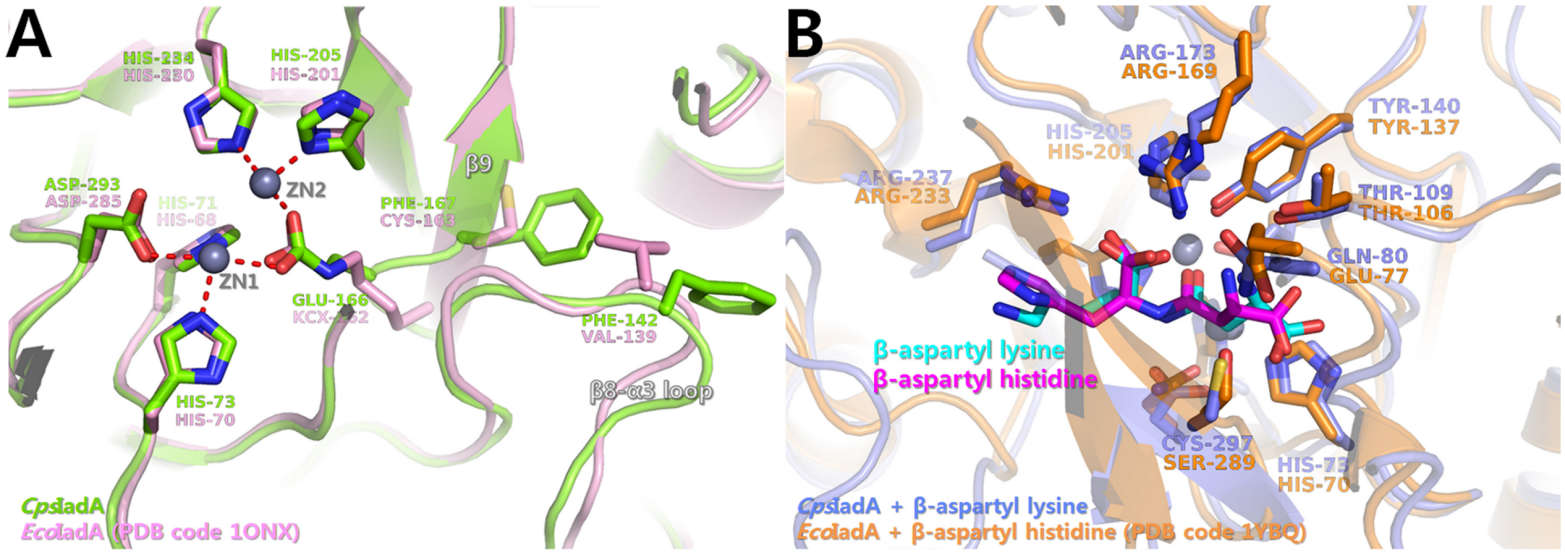
Structural superposition between *Cps*IadA and *Eco*IadA. (A) Structural comparison between ligand-free *Cps*IadA (green) and *Eco*IadA (PDB code 1ONX; pink) at the active site. (B) Structural comparison between β-isoaspartyl lysine (cyan), bound *Cps*IadA (slate blue), and β-isoaspartyl histidine (magenta) bound *Eco*IadA (PDB code 1YBQ; orange) at the active site. Two Zn ions in *Cps*IadA are represented by grey spheres; the Zn ions in *Eco*IadA have been removed for clarity in both Fig 5A and Fig 5B.

A structural comparison between the ligand-free wild type *Cps*IadA structure and the structure of the *Cps*IadA E80Q mutant complexed with β-isoaspartyl lysine (Fig 3A and 3B) show several structural changes occur upon ligand binding. Notably, upon substrate binding, a slight tilt of residue Tyr140 is observed with a 61° rotation. Tyr140 interacts with the O04 and O07 atoms of the bound β-isoaspartyl lysine (Fig 4A and 4C). The activity assay revealed that the Y140F mutant of *Cps*IadA had a significant reduction rate of catalysis confirming that this tyrosine residue is important for enzymatic catalysis by *Cps*IadA. In previous studies, the *Eco*IadA Y137F mutant (corresponding to Tyr140 in *Cps*IadA) (PDB code 2AQV) biochemical analysis suggested that the phenolic hydroxyl group in Tyr137 might function as a Lewis acid catalyst interacting with the reaction intermediate [31, 32]. The bound β-isoaspartyl lysine, with the exception of the lysine side-chain region, forms tight interactions with *Cps*IadA. In detail, the O07 atom of the bound β-isoaspartyl lysine interacts with the ZN1 metal ion. The OG1 atom of Thr109 and the OE1 atom of Glu80 interact with the N11 atom of the β-isoaspartyl lysine, and the SG atom of Cys297 interacts with the O13 atom of β-isoaspartyl lysine. Residues Arg173 and Arg237 interact with the O01 and O04 atoms of the β-isoaspartyl lysine. As noted above, the lysine side chain of β-isoaspartyl lysine did not have any specific interactions with *Cps*IadA. Thus, this region has a relatively weak electron density (Figs 3B and 4B). These results suggest that the side chain of the second amino acid in β-the isoaspartyl substrate is not critical for ligand recognition and specificity. They are also in good agreement with our activity assays that showed that *Cps*IadA has a broad substrate specificity with respect to the second amino acid position in the β-isoaspartyl substrate (Table 2). In addition, a structural comparison between the β-isoaspartyl lysine complexed *Cps*IadA and the β-isoaspartyl histidine complexed *Eco*IadA (PDB code 1YBQ) revealed that bound substrates have a similar conformation with the exception of the second amino acid side chain [14]. As noted above, the biggest difference between *Cps*IadA and the *Eco*IadA structure is in the nature of the Zn ion stabilizing residue. The Glu166 residue of *Cps*IadA directly interacts with the Zn ions, however in *Eco*IadA, carbamylated lysine 162 interacts with the Zn ions. Structural superposition of *Cps*IadA onto *Eco*IadA (PDB code 1YBQ) also showed that the β12-α8 loop region (residues 165–166) in *Cps*IadA protruded into the active site to interact with Zn metal ions. Because of this, although the Glu166 side chain length is shorter than that of carbamylated lysine, the glutamate side chain could directly interact with the Zn^2+^ ion in *Cps*IadA (Fig 5).

**Table 2:** Steady-state kinetic parameters of *Cps*IadA.

.Substrate	*k*_cat_ (s^-1^)	*K*_m_ (mM)	*k*_cat_/*K*_m_ (M^-1^ s^-1^)
β-Asp-Leu	164 ± 5.4	0.71 ± 0.08	(2.3 ± 0.3) x 10^5^
α-Asp-Leu	89 ± 2.5	6.9 ± 0.4	(1.3 ± 0.8) x 10^4^
β-Asp-Gly	181 ± 7.0	4.7 ± 0.4	(3.8 ± 0.4) x 10^4^
β-Asp-Ala	166 ± 13	1.2 ± 0.3	(1.4 ± 0.3) x 10^5^
β-Asp-Phe	145 ± 6.5	0.49 ± 0.09	(3.0 ± 0.5) x 10^5^
β-Asp-Lys	256 ± 6.1	1.1 ± 0.09	(2.3 ± 0.2) x 10^5^
β-Asp-His	74 ± 3.6	5.3 ± 0.5	(1.4 ± 0.2) x 10^4^
β-Ala-Ala	-	0.93 ± 0.3	(1.0 ± 0.4) x 10^1^

### Biochemical characteristics

**Fig 6:**
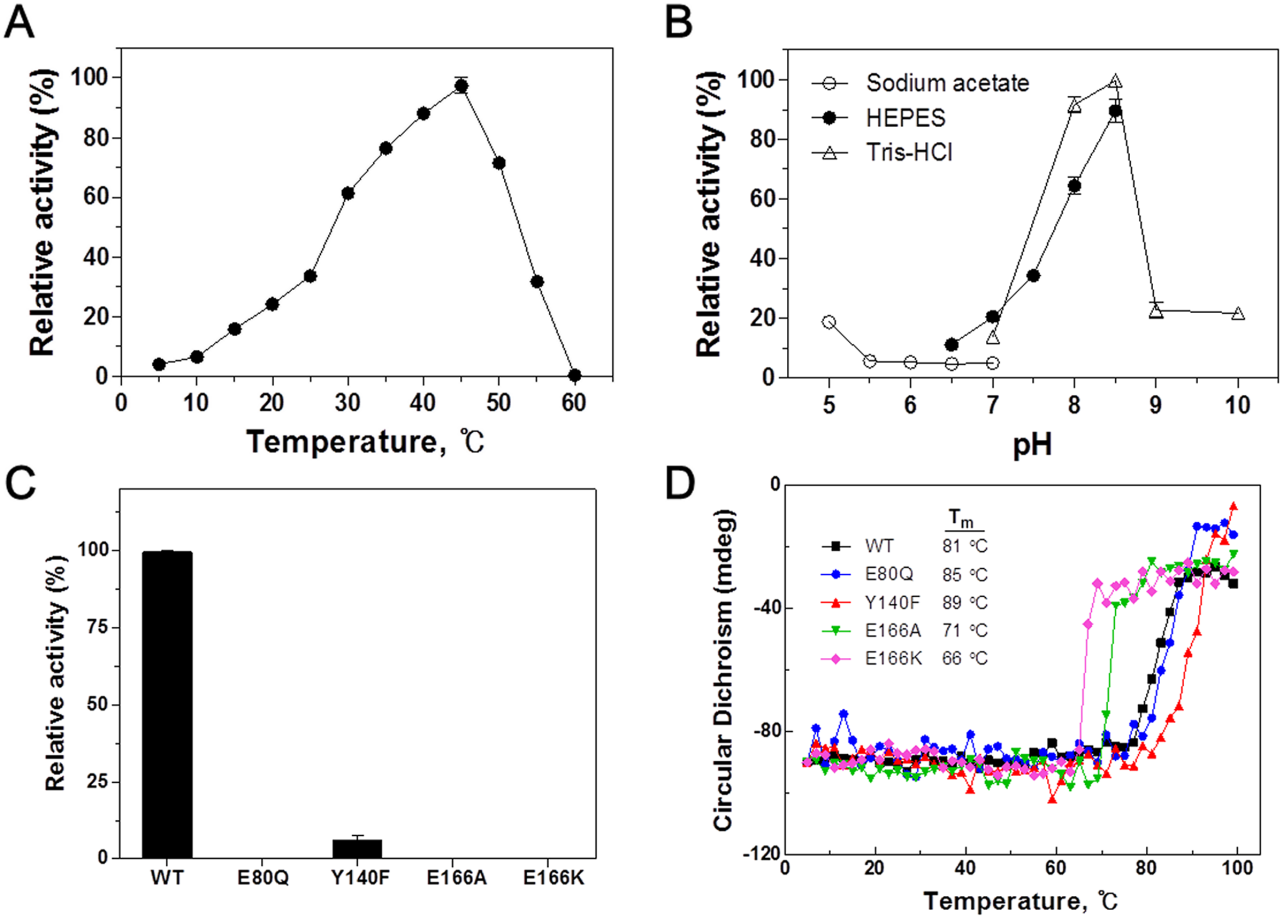
Biochemical characterization of *Cps*IadA. (A) Effect of temperature on *Cps*IadA activity. The relative β-Asp-Leu hydrolytic activity of purified *Cps*IadA at different temperatures (5–60°C) was determined at pH 8.0 in 0.1 M HEPES buffer. The activity at the optimal temperature was set as 100%. (B) Effect of pH on *Cps*IadA activity. The pH dependence for hydrolysis of β-Asp-Leu by *Cps*IadA was measured at 30°C. The activity at the optimal pH was set as 100%. (C) Hydrolysis of β-Asp-Leu by wild-type and *Cps*IadA mutants. The effects of single mutations on *Cps*IadA activity was investigated using the active site mutants E80Q, Y140F, E166A, and E166K. Reactions were performed under standard assay conditions and the activity of the wild-type enzyme using β-Asp-Leu as a substrate was taken as 100%. (D) Changes in circular dichroism spectra during thermal denaturation of wild-type and *Cps*IadA mutants. CD spectra were measured over a temperature range of 5–99°C at 222 nm.

The effect of temperature on the catalytic activity of *Cps*IadA was determined by incubating the enzyme at various temperatures in the presence of 10 mM β-Asp-Leu as the substrate (Fig 6A). Under these assay conditions, *Cps*IadA exhibited a maximum activity at 45°C and complete inactivation was observed at 60°C. The thermal stability of *Cps*IadA was also measured. The denaturation temperature (T_m_) for CpsIadA was determined to be 81°C (Fig 6D). Although *Cps*IadA was isolated from the psychrophilic bacteria, *Colwellia psychrerythraea*, this enzyme does not show characteristics typical of psychrophilic enzymes. Our findings suggest that *Cps*IadA is a thermostable enzyme, which may be attributed to its high-order oligomerization. Larger oligomeric states, such as seen here, have been observed in some hyper-thermostable proteins when compared to their mesophilic counterparts [33, 34]. It has been proposed that higher order oligomerization, along with a stronger ion-pairing network and additional disulfide bridges are structure-stabilizing factors that contribute to a high thermal stability [33–36]. The isoaspartyl dipeptidase activity of *Cps*IadA was pH-dependent, with a maximum activity at pH 8–8.5, as shown in Fig 6B. When the pH was either decreased or increased beyond this range the enzyme activity dropped off sharply.

### Substrate specificity

To investigate the substrate specificity of *Cps*IadA, six β-aspartyl dipeptides (β-Asp-Leu, β-Asp-Gly, β-Asp-Ala, β-Asp-Phe, β-Asp-Lys, and β-Asp-His) were evaluated as substrates, in addition to α-Asp-Leu and β-Ala-Ala (Table 2). Typical Michaelis-Menten curves were obtained for *Cps*IadA with all β-aspartyl dipeptides tested. Although β-Asp-Leu is the best substrate for *Eco*IadA, *Cps*IadA displayed the highest catalytic efficiencies (*k*_cat_/*K*_m_) for β-Asp-Phe (3.0 x 10^5^ M^-1^ s^-1^) followed by β-Asp-Leu and β-Asp-Lys. However, the *k*_cat_ is greater for β-Asp-Lys than β-Asp-Phe with values of 256 and 164 s^-1^, respectively. In contrast to *Eco*IadA, which had a two or three-orders of magnitude lower *k*_cat_/*K*_m_ for β-Asp-Gly compared to other tested β-aspartyl dipeptides, no such significant reduction in activity for β-Asp-Gly was observed for *Cps*IadA [14].

When α-Asp-Leu was tested as a substrate *Cps*IadA also exhibited dipeptidase activity with a *k*_cat_/*K*_m_ of 1.4 x 10^4^ M^-1^ s^-1^, indicating that the shift of the α-amino group of aspartate moiety between C_2_ and C_3_ within substrates causes only slight reduction in enzyme activity. On the other hand, β-Ala-Ala was not an efficient substrate for *Cps*IadA, demonstrating that the α-carboxylate group of a β-aspartyl dipeptide is essential for enzyme activity. These results are consistent with those seen for *Eco*IadA. Overall, only slightly differences in substrate specificity were found for *Cps*IadA compared with *Eco*IadA, despite the different active site residue composition and substrate binding pocket [14].

### Mutational studies of *Cps*IadA

We investigated the effect of active site mutations on the catalytic activity and structural integrity of *Cps*IadA. Four active site mutants (E80Q, Y140F, E166A, and E166K) were constructed and their enzymatic activity was assessed. In addition, their denaturation temperatures (T_m_) values were measured using circular dichroism (CD) spectroscopy (Fig 6C and 6D). The E80Q mutant had no catalytic activity toward β-Asp-Leu, but its CD spectra and denaturation temperature (T_m_) were similar to WT, indicating that this mutation affected catalytic activity but not the overall folding and integrity of *Cps*IadA. In the case of the Y140F mutant, less than 10% catalytic activity was observed toward β-Asp-Leu and the CD spectra was not significantly different to the WT. Notably, unlike the WT and the other mutants Y140F had a tendency to aggregate during purification and storage, but soluble Y140F was the most thermally stable. Glu166 interacts with Zn in the active site and was substituted by mutagenesis with either Ala or Lys, an equivalent residue in *E*.*coli*. Both the E166A and E166K mutants exhibited a complete loss of activity and a considerable decrease in melting temperature. These results were probably due inhibition of Zn binding to the *Cps*IadA active site caused by mutation of Glu166, clearly demonstrating that Glu166 is critical for the catalytic activity and structural integrity of *Cps*IadA.

### Phylogenetic analysis of *Cps*IadA

To investigate the sequence diversity and evolutionary characteristics of IadA enzymes, individual sequences were aligned and a phylogenetic tree was constructed with a total of thirty-nine IadA amino acid sequences from bacteria and archaea (Fig 1 and S3 Fig). As a result, the IadA enzymes could be classified into two groups (Type I and II) based on the active site residue composition. Type I IadA enzymes have a glutamate for metal binding in the active site, whereas Type II IadA enzymes have a post-translationally carbamylated-lysine residue. Thus, *Cps*IadA, having Glu166, is a Type I IadA and *Eco*IadA, having carbamylated-Lys162, is a Type II IadA. Interestingly, bacteria in the order *Enterobacteriales* (class *Gammaproteobacteria*, phyla *Proteobacteria*) including *E*. *coli* and *Salmonella* are Type II IadAs, whereas all the other bacteria and archea are type I IadAs. From these data it can be hypothesized that Type II IadA enzymes have evolved in the direction of allowing post translational modification of the amino acid residue in the active site for metal binding and catalytic activity.

In conclusion, the crystal structure of *Cps*IadA presented here shows strong sequence and structural similarity to the *Eco*IadA but it has a different active-site residue configuration, using Glu166 instead of a carbamylated lysine residue. Interestingly, this difference is a hallmark of IadA proteins and it can be used to separate bacteria and archea into two groups (Type I and II). In *Eco*IadA, the carbamylation of lysine 162 residue requires a carbonate supply and time for this maturation step to occur, whereas these are not needed for *Cps*IadA. Thus, type I and II IadAs may have different regulatory mechanisms for controlling their activation in order to remove isodipeptides *in vivo*. However, it is still unclear why IadAs have evolved into two different types. Further analysis is therefore required to fully clarify the relationship between the structure and biological function of type I and type II IadAs.

## Supporting information

S1 Fig X-ray fluorescence spectrum (blue line) was measured using single unliganded *Cps*IadA crystal at the BL-5C of the Pohang Accelerator Laboratory (Pohang, Korea). This spectrum shows a clear absorption edge at the zinc peak. Thus, this result allows us to confirm the presence of zinc ions in *Cps*IadA structure. The X-axis indicates X-ray photon energy expressed in kiloelectron volt unit.
(TIF)

S2 Fig Effects of divalent zinc ions on the activity of *Cps*IadA. The activity of *Cps*IadA was determined in the absence and presence of ZnCl_2_ under standard assay conditions. The metal-free *Cps*IadA was prepared by 10 mM EDTA treatment for 3 hours at room temperature, and then EDTA was removed by dialysis against 20 mM Tris-HCl buffer (pH 8.0) with 150 mM NaCl. Relative activities were measured under standard assay conditions and the activity of native *Cps*IadA was defined as 100%. All measurements were performed in triplicate.
(TIF)

S3 Fig Phylogenetic relationships between *Cps*IadA protein sequences and their bacterial and archaeal homologues. (A) The phylogenetic tree was drawn based on a multiple sequence alignment of *Cps*IadA homologues from a range of species representing different phyla. The alignment of the amino acid sequences was performed using ClustalW software. The evolutionary history was inferred using the Neighbor-Joining method [37]. The bootstrap consensus tree inferred from 1000 replicates is taken [38]. The evolutionary distances were computed using the JTT matrix-based method [39]. Evolutionary analyses were conducted in MEGA7 [30]. (B) To estimate the contribution of the active site Glu/Lys residue in classifying different enzyme types, the Lys in Type II IadAs was mutated to Glu, and then the phylogenetic tree was constructed. Sequences are named according to their species identity along with the NCBI accession number. The *Cps*IadA sequence is marked with a black dot, and a cluster with sequences of Type II IadAs are marked with a red box.
(TIF)
