# Supplementary figures and images for: Crystal structure and functional characterization of an isoaspartyl dipeptidase (CpsIadA) from Colwellia psychrerythraea strain 34H
Source: PLoS One. 2017 Jul 19;12(7):e0181705. doi: 10.1371/journal.pone.0181705 (PMC5517026; doi:10.1371/journal.pone.0181705)

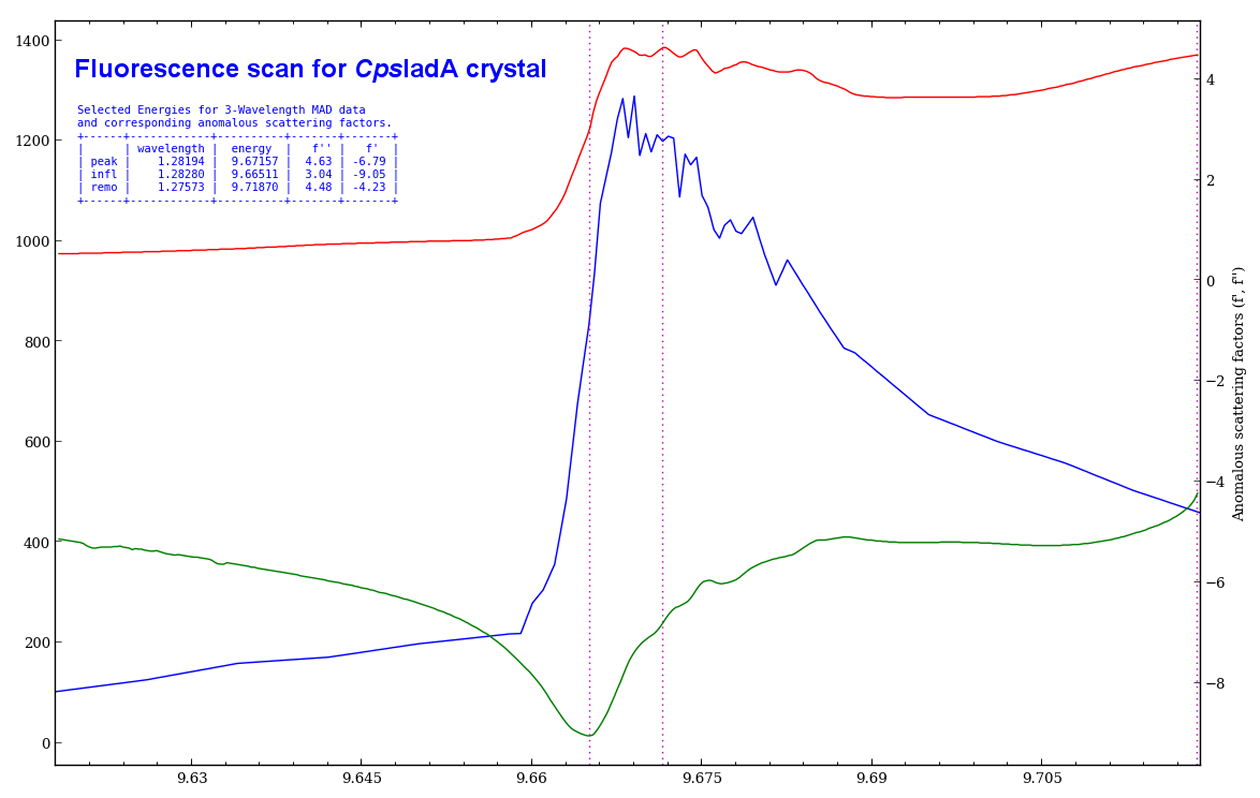

Supplement: S1 Fig — This spectrum shows a clear absorption edge at the zinc peak. Thus, this result allows us to confirm the presence of zinc ions in CpsIadA structure. The X-axis indicates X-ray photon energy expressed in kiloelectron volt unit. (TIF) [file pone.0181705.s001.tif]

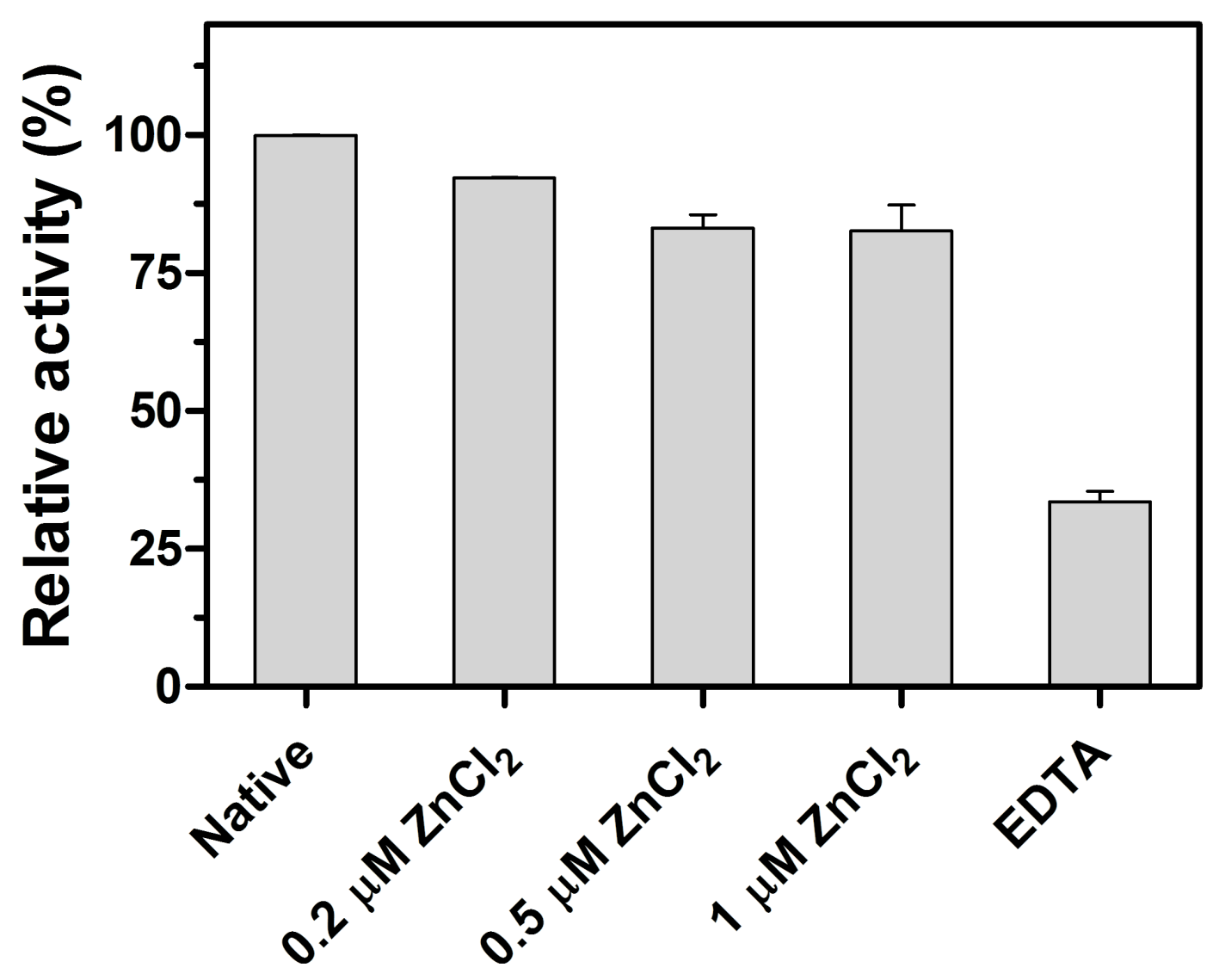

Supplement: S2 Fig — The activity of CpsIadA was determined in the absence and presence of ZnCl2 under standard assay conditions. The metal-free CpsIadA was prepared by 10 mM EDTA treatment for 3 hours at room temperature, and then EDTA was removed by dialysis against 20 mM Tris-HCl buffer (pH 8.0) with 150 mM NaCl. Relative activities were measured under standard assay conditions and the activity of native CpsIadA was defined as 100%. All measurements were performed in triplicate. (TIF) [file pone.0181705.s002.tif]

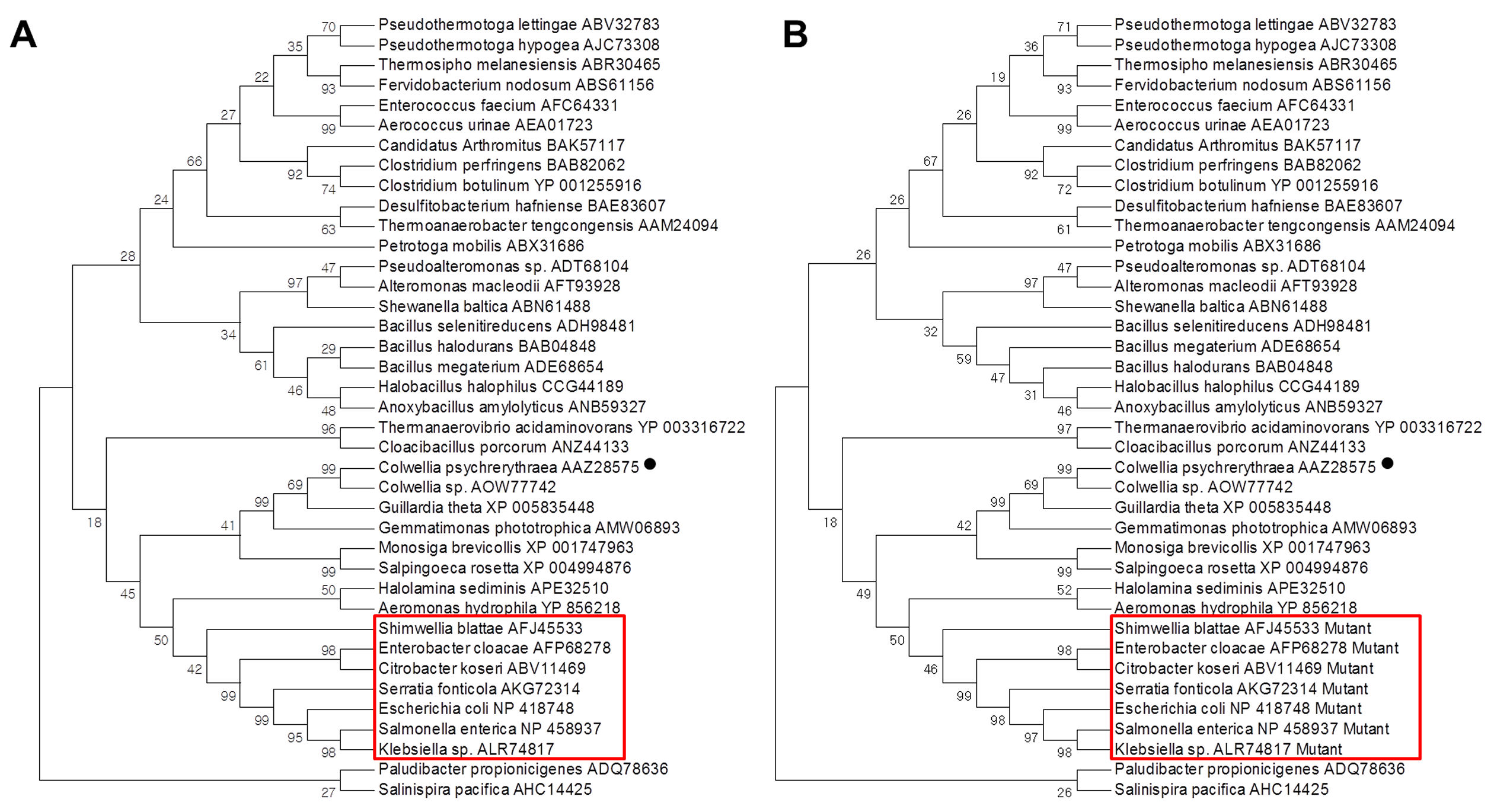

Supplement: S3 Fig — (A) The phylogenetic tree was drawn based on a multiple sequence alignment of CpsIadA homologues from a range of species representing different phyla. The alignment of the amino acid sequences was performed using ClustalW software. The evolutionary history was inferred using the Neighbor-Joining method [37]. The bootstrap consensus tree inferred from 1000 replicates is taken [38]. The evolutionary distances were computed using the JTT matrix-based method [39]. Evolutionary analyses were conducted in MEGA7 [30]. (B) To estimate the contribution of the active site Glu/Lys residue in classifying different enzyme types, the Lys in Type II IadAs was mutated to Glu, and then the phylogenetic tree was constructed. Sequences are named according to their species identity along with the NCBI accession number. The CpsIadA sequence is marked with a black dot, and a cluster with sequences of Type II IadAs are marked with a red box. (TIF) [file pone.0181705.s003.tif]
